# Safety and feasibility study of uniportal video‐assisted thoracoscopic pulmonary wedge resection without postoperative chest tube drainage: a retrospective propensity score‐matched study

**DOI:** 10.1093/icvts/ivad196

**Published:** 2023-12-13

**Authors:** Qingjie Yang, Shenghua Lv, Qingtian Li, Linhui Lan, Xiaoyan Sun, Xinhai Feng, Kaibao Han

**Affiliations:** Department of Thoracic Surgery, Xiamen Humanity Hospital of Fujian Medical University, Xiamen, China; Department of Thoracic Surgery, Xiamen Humanity Hospital of Fujian Medical University, Xiamen, China; Department of Thoracic Surgery, Xiamen Humanity Hospital of Fujian Medical University, Xiamen, China; Department of Thoracic Surgery, Xiamen Humanity Hospital of Fujian Medical University, Xiamen, China; Department of Thoracic Surgery, Xiamen Humanity Hospital of Fujian Medical University, Xiamen, China; Department of Thoracic Surgery, Xiamen Humanity Hospital of Fujian Medical University, Xiamen, China; Department of Thoracic Surgery, Xiamen Humanity Hospital of Fujian Medical University, Xiamen, China

**Keywords:** Pulmonary wedge resection, Drainage, Propensity score matching, Video-assisted thoracic surgery, Chest tube

## Abstract

**OBJECTIVES:**

The aim of this study was to assess the impact of postoperative chest tube drainage (CTD) on safety and postoperative recovery by comparing patients with pulmonary nodule undergoing uniportal video‐assisted thoracoscopic pulmonary wedge resection with and without postoperative CTD.

**METHODS:**

We retrospectively analysed the data of patients who underwent video‐assisted thoracoscopic pulmonary wedge resection for pulmonary nodule at our hospital between 2018 and 2022. In cases where a 12-Fr chest tube was used following the procedure, the tube was not usually removed until the day after surgery. Therefore, the eligible patients were categorized into the drainage tube or the no‐drainage tube group according to the use of postoperative CTD. Propensity score matching at a ratio of 1:1 was performed using clinicopathologic and demographic variables. The highest postoperative pain score, postoperative complication rate, postoperative length of stay and hospitalization costs were compared between the 2 groups.

**RESULTS:**

A total of 275 eligible patients, including 150 and 125 patients in the drainage tube and no‐drainage tube groups, respectively, were included in the study. After propensity score matching, there were 102 patients in each group. The postoperative complication rate during hospitalization and at 1 week and 1 month after discharge were not significantly different between the 2 groups (*P* > 0.05 for all). The highest postoperative pain score was significantly lower in the no‐drainage tube group than in the drainage tube group [2.02 (standard deviation: 0.81) days vs 2.31 (standard deviation: 0.76) days, *P* = 0.008]. The postoperative length of stay was significantly shorter in the no‐drainage tube group than in the drainage tube group {3.00 [interquartile ranges (IQRs): 2.00–4.00] days vs 2.00 (IQRs: 1.00–3.00) days, *P* < 0.001}. Similarly, the total hospitalization costs were significantly lower in the no‐drainage tube group than in the drainage tube group [33283.74 (IQRs: 27098.61–46718.56) yuan vs 26598.67 (IQRs: 22965.14–29933.67) yuan, *P* < 0.001].

**CONCLUSIONS:**

Omission of postoperative CTD was safe and feasible in patients with pulmonary nodule undergoing wedge resection. The no-postoperative-drainage policy can substantially shorten the length of hospital stay and reduce the postoperative pain and hospitalization costs without increasing the risk of postoperative complications.

**Figure:**
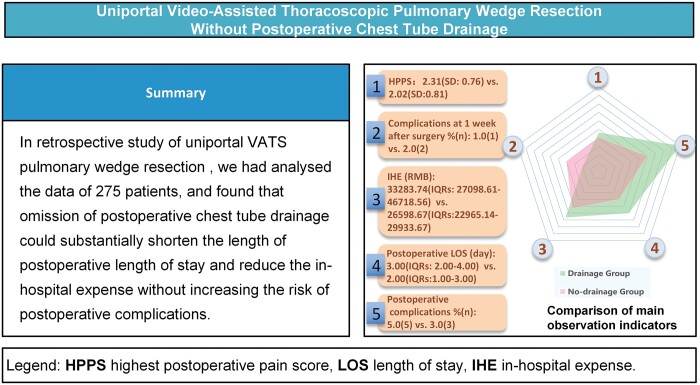

## INTRODUCTION

Postoperative chest tube drainage (CTD) is a routine procedure to drain air, fluid and blood from the thoracic cavity following pulmonary surgery and to observe for the presence of active bleeding and air leaks. However, this procedure has several notable side effects such as increased pain intensity, limited expectoration and pulmonary re‐expansion, reduced functional recovery and prolonged hospital stay [1–5]. Advances in video‐assisted thoracic surgery and improvements in minimally invasive surgical instruments such as cutting staplers have allowed surgeons to create smaller incisions to minimize the risk of postoperative intrathoracic bleeding and air leaks and to reduce postoperative complication rates to below 1%, especially during straightforward procedures such as thoracoscopic pulmonary wedge resection [6]. In thoracic surgery, questions remain primarily regarding the need for postoperative CTD for simple thoracic surgeries such as thoracoscopic pulmonary wedge resection and whether this routine procedure may benefit postoperative recovery. Clinical studies [7–18] comparing post‐thoracic surgical performance and the omission of postoperative CTD have unanimously demonstrated that recovery after surgery is improved in patients who receive either a small‐bore chest tube (10 or 12-Fr pigtail drain) or no chest tube. Conversely, studies have noted that omitting postoperative CTD is inevitably associated with a potential risk of leaving air or fluid in the thoracic cavity after thoracic surgery, which interferes with the subsequent observation of postoperative intrathoracic bleeding and air leaks [19].

In the present retrospective study, we aimed to analyse the relationship between postoperative CTD and postoperative recovery in patients who underwent uniportal video‐assisted thoracoscopic pulmonary wedge resection (U‐VATS wedge). Therefore, we determined whether postoperative recovery differed between the patients in whom short-term postoperative CTD was used and those in whom postoperative CTD was omitted. In patients with postoperative CTD, a 12-Fr pigtail drain was left in place until the morning after surgery to achieve adequate drainage of air and fluid from the thoracic cavity, to closely observe potential intrathoracic bleeding and air leaks and to minimize the potential risks associated with postoperative CTD.

This article is written following STROBE checklist.

## METHODS

### Ethics statement

This study was approved by the Medical Ethics Committee of Xiamen Humanity Hospital of Fujian Medical University (No. HAXM-EMC-20221017-001-01). This study was in accordance with the provisions of the Declaration of Helsinki. Considering its retrospective design, the requirement of informed consent of each patient was waived by the ethics committee.

### Cases collected

The study cohort included patients with pulmonary nodule who were admitted to the Xiamen Humanity Hospital between 1 August 2018 and 31 December 2022 and underwent U‐VATS wedge. The inclusion criteria were U‐VATS wedge with double‐lumen tubes under general anaesthesia and unilateral wedge resection of solitary or multiple peripheral pulmonary nodules. The exclusion criteria included visceral pleural damage caused by the removal of severe pleural adhesions, concomitant emphysema or pulmonary infection, perioperative use of anticoagulants, segmentectomy or lobectomy indicated by intraoperative histopathology, pulmonary hilar or mediastinal lymph node sampling or dissection. These conditions would increase the risk of massive exudation in the surgical area and pulmonary air leakage after surgery, needed postoperative CTD. In addition, patients who met the discharge criteria still required a prolonged hospital stay were also excluded. In the present study, the eligible patients were categorized into the drainage tube (DT) and no‐drainage tube (NT) groups based on the use of postoperative CTD.

### Uniportal video‐assisted thoracoscopic pulmonary wedge resection

#### Surgical procedures

**Figure 1:**
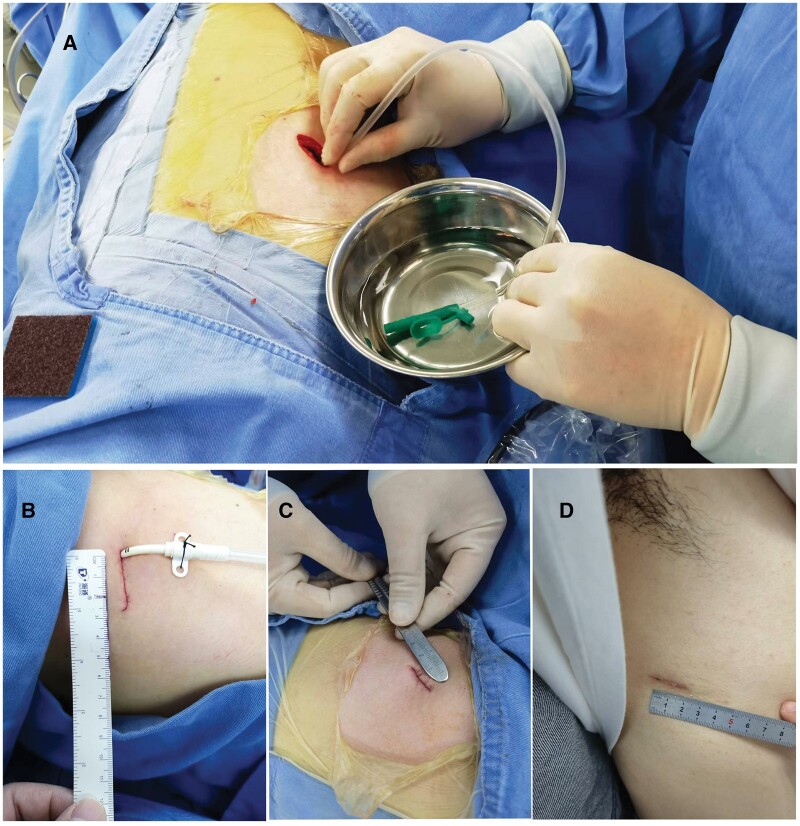
Exhaust the air in chest during operation and surgical incision. (**A**) Exhaust the air in chest during operation; (**B**) surgical incision for drainage tube placement; (**C**) surgical incision without drainage tube; and (**D**) incision at 1 month after surgery.

Preoperative CT images were used to determine whether the location of pulmonary nodules was easy and accurate during the operation. The pulmonary nodules that were difficult to locate during the operation were sent to the CT room for percutaneous methylene blue localization or percutaneous hookwire localization before the operation. A double‐lumen tube was inserted under general anaesthesia. The patient was placed in a supine position, turned 90° to the unaffected side and prepped and draped in the standard sterile fashion. For nodules in the upper lobe, an incision was made in the fourth intercostal space along the midaxillary line. For nodules in the middle and lower lobes, the site of entry was above the fifth intercostal space in the midaxillary line. In all cases, only 1 incision, 2–3 cm in width, was made using an electrotome, and a wound retractor was used to create a working port. Next, the thoracoscope was introduced into the chest. The lung tissue at the lesion site was lifted with grasping forceps, and the lesion was resected with 2-cm margins using a linear cutting stapler (45/60 cm blue cartridge, Shanghai Yisi Medical Technology Co., LTD, China) from the bottom. Fresh-frozen specimens of the resected lung tissue were examined under microscopy immediately after the resection. No further procedures were necessary in cases where the pathologic examination suggested adequate wedge resection, ie benign features, atypical adenomatoid hyperplasia, carcinoma *in situ* or microinvasive carcinoma with a negative margin. In patients categorized in the DT group, a 12-Fr chest tube (ABLE drainage catheter, Guangdong Baihe Medical Technology Co. LTD, China) was inserted before suturing the wound layers. In patients categorized in the NT group, a 16-G suction cannula was inserted through the incision for pleural air drainage. Interrupted sutures were placed on the chest wall muscles and the layers adjacent to both sides of the cannula without tying the knots. Then, the free end of the cannula was placed in a cup of water, and the anaesthetist expanded the operated lung to mechanically drain the pleural air via the cannula. When the bubbling of air out of the cannula stopped, vacuum aspiration with the cannula was initiated while slowly retracting the cannula from the chest. Sutures close to the cannula were immediately tied following the completion of the retraction. Finally, the wound layers were sutured. See Fig. 1.

In the early stage, chest tube was always placed during our surgeries. Later on, we gradually discovered that omitting chest tube was safe, so we started to place chest tube less and less until it became routine not to place chest tube. It should be noted that prior to surgery, we inform patients in the surgical consent form and preoperative consultation that the decision to place or not place a chest drainage tube will be made based on individual circumstances during the operation.

### Postoperative management

After the surgery, the patients were transferred to the ward after anaesthesia recovery and extubation in the postanaesthesia care unit. Analgesics were routinely administered and included 40 mg parecoxib in 100 ml of 0.9% sodium chloride as an intravenous drip, twice a day. In patients with unbearable pain, 100 mg tramadol was intramuscularly administered for temporary relief.

In the NT group, ∼6 h after the patient’s return to the ward, bedside chest direct digital radiograph (DR) examination was performed with the patient in a sitting position. The DR images were evaluated for the following next steps. Patients with no DR findings of pleural effusion or pneumothorax were discharged the morning after surgery. In patients with DR findings of pleural effusion in the operated lung, where the fluid reached above the level of the upper portion of the diaphragm, and in those with >20% pneumothorax requiring closed drainage using a 12-Fr chest tube placed in the seventh intercostal space (for pleural effusion) along the posterior axillary line or in the second intercostal space (for pneumothorax) in the midclavicular line, the chest tube was removed after the confirmation of the absence of air in the chest tube, <200 ml of daily drainage, and no DR signs of pleural effusion or pneumothorax. In patients with DR findings of pleural effusion on the operated side, where the fluid was below the level of the upper portion of the diaphragm, and in those with <20% pneumothorax, another chest DR scan was performed the next morning. The patients without worsening symptoms were discharged, and the abovementioned closed drainage was performed if the DR findings suggested otherwise.

In the DT group, bedside chest DR was performed with the patient in a sitting position on the morning of the postoperative hospital stay 1. The DR images were evaluated for the following potential outcomes. In patients with no DR signs of pleural effusion or pneumothorax, no air leakage from the chest tube, and a total drainage volume of <200 ml on the day of surgery, the chest tube was removed, and the patient was discharged the same day or on the morning of postoperative hospital stay 2. In patients with DR findings of pleural effusion or pneumothorax requiring postural drainage to remove fluid or air from the thoracic cavity, another chest DR scan was scheduled for the day after postural drainage. In patients with air leaks or fluid drainage from the chest tube and in those with a total fluid drainage volume of >200 ml on the day of surgery, the CTD was continued.

Follow‐up chest DR scans were performed 1 week after the surgery to evaluate for pneumothorax, pleural effusion and pulmonary infection. Closed drainage was performed in patients with DR findings indicating >30% pneumothorax or pleural effusion where the fluid was above the level of the upper portion of the diaphragm. In patients with <30% pneumothorax or a pleural effusion below the level of the upper portion, another chest DR scan was performed 3 days later to determine whether closed drainage was necessary per the abovementioned criteria.

Follow‐up chest CT scans were performed at postoperative 1 month to assess pulmonary recovery.

### Observation indicators

The following preoperative patient data were collected as baseline characteristics: age, sex, forced expiratory volume in 1 s (FEV1), maximal voluntary ventilation (MVV), cardiac ejection fraction (EF), pulmonary nodule size, number of simultaneously resected pulmonary nodules and pulmonary nodule location. Intra‐ and postoperative data included primary observation indicators: highest postoperative pain score (POPS), postoperative length of stay (LOS), complications, hospitalization costs, postoperative relapse of pleural effusion or pneumothorax. Secondary observation indicators: surgical duration, intraoperative bleeding, total postoperative drainage, drainage duration, postoperative histopathology and follow‐up findings at 1 week and 1 month after surgery.

Pulmonary nodule size was defined as the maximum axial diameter of the pulmonary nodule. Surgical duration was defined as the time period between the start of the first incision and the suturing of overlying skin, including the examination of frozen sections, which lasted ∼30 min. Total postoperative drainage was defined as the total volume of fluid drained from the thoracic cavity during the interval between the end of surgery and chest tube removal, which was only applicable to the DT group. Highest POPS was defined as the highest pain score obtained from multiple assessments during the interval between the end of surgery and discharge; the assessments were performed twice daily and during each episode of intense pain using a visual analogue scale. Postoperative complications included persistent air leaks, active intrathoracic bleeding, pulmonary infection and cardiac insufficiency. In patients with multiple pulmonary nodules, the postoperative histopathologic evaluation of the most severe lesion was included in the present study.

### Statistical analysis

SPSS version 26.0 (IBM Corp, USA) was used for all statistical analyses. Propensity score matching (PSM) was performed to reduce confounding effects, and the patients were matched at a ratio of 1:1 with a calliper of 0.05. The following indicators were included in the PSM: age, sex, preoperative lung and heart function parameters (FEV1, MVV and EF), pulmonary nodule size, number of simultaneously resected pulmonary nodules, pulmonary nodule location, surgical duration, intraoperative bleeding and postoperative histopathologic findings.

Normally distributed data were presented as means [standard deviation (SD)], while non-normally distributed data were represented by the medians [interquartile ranges (IQRs): first quartile–third quartile]. Categorical data were presented as numbers with percentages.

For covariates before matching, normally distributed continuous data were analysed using independent-samples *t* test. Non-normally distributed continuous data were analysed using the independent samples Wilcoxon rank-sum test. Categorical data were analysed using the chi-square test or Fisher's exact test, which was used when the expected frequency was <5%.

For covariates after matching, normally distributed continuous data were analysed using paired samples *t* test. Non-normally distributed continuous data were analysed using the paired samples Wilcoxon rank-sum test. Categorical data were still analysed using the chi-square test or Fisher's exact test.

All statistical analyses were conducted with an α cut-off value of 0.05.

## RESULTS

### Intergroup comparison of pre‐ and intraoperative clinical characteristics before and after PSM

**Figure 2:**
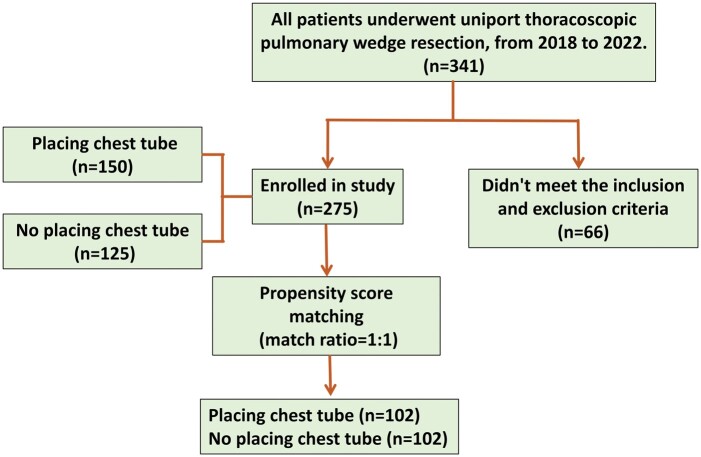
Cases collected.

A total of 341 patients underwent the U‐VATS wedge between 1 August 2018 and 31 December 2022, and 275 patients met the inclusion and exclusion criteria. The 275 patients were assigned to the DT (*n* = 150) or the NT (*n* = 125) group. Among the evaluated indicators, number of simultaneously resected pulmonary nodules, maximum lesion diameter, surgical duration and intraoperative bleeding were significantly different between the DT and NT groups (*P *>* *0.05 for all). The PSM identified 102 matched pairs with similar clinical characteristics, with no significant differences between the 2 groups. See Fig. 2 and Table 1.

**Table 1:** Clinical characteristics of the DT and NT groups (before and after propensity score matching)

Characteristics	Before propensity score matching					After propensity score matching				
	DT group (*n* = 150)	NT group (*n* = 125)	SMDs	*t*/Z/χ^2^	*P*-value	DT group (*n* = 102)	NT group (*n* = 102)	SMDs	*t*/Z/χ^2^	*P*-value
Sex, % (*n*)			0.251	3.013	0.083			0.141	1.316	0.251
Male	30.0 (45)	20.8 (26)				27.5 (28)	20.6 (21)			
Female	70.0 (105)	79.2 (99)				72.5 (74)	79.4 (81)			
Age (years)	49.07 (13.99)	46.63 (1.79)	0.257	1.542	0.124	47.52 (13.25)	47.03 (11.61)	0.039	−0.281	0.779
Pulmonary function										
Actual FEV1/pre-FEV1 (%)	101.05 (12.34)	101.72 (15.66)	0.009	−0.393	0.695	101.48 (13.73)	100.91 (11.65)	0.038	0.268	0.789
Actual MVV/pre-MVV (%)	96.96 (16.29)	98.62 (15.03)	0.132	−0.867	0.387	97.19 (16.18)	99.24 (15.23)	0.131	0.932	0.352
EF(%)	67.07 (3.94)	67.63 (4.03)	0.232	−1.117	0.242	67.13 (4.02)	67.04 (3.91)	0.022	−0.159	0.874
Number of simultaneously resected pulmonary nodules (*n*)	1.31 (0.79)	1.04 (0.27)	0.012	−2.678	0.008	1.16 (0.36)	1.05 (0.28)	0.002	−1.905	0.059
Maximum lesion diameter (mm)^a^	9.00 (7.10–12.80)	8.30 (6.45–10.60)	0.205	−2.316	0.021	8.50 (6.60–11.50)	7.85 (6.40–11.20)	0.038	−0.435	0.664
Pulmonary nodule location, % (*n*)			0.336	8.158	0.086			0.027	5.449	0.244
Right upper lobe	27.3 (41)	38.4 (48)				33.3 (34)	35.3 (36)			
Right middle lobe	5.3 (8)	5.6 (7)				3.9 (4)	3.9 (4)			
Right lower lobe	27.3 (41)	14.4 (18)				22.5 (23)	14.7 (15)			
Left upper lobe	25.3 (38)	32.0 (40)				24.5 (25)	36.3 (37)			
Left lower lobe	14.7 (22)	9.6 (12)				15.7 (16)	9.8 (10)			
Pathological types % (*n*)			0.199	4.895	0.298			0.008	1.353	0.852
AAH	11.3 (17)	8.0 (10)				9.8 (10)	6.9 (7)			
AIS	36.7 (55)	45.6 (57)				40.2 (41)	46.1 (47)			
MIA	16 0.0 (24)	16.8 (21)				16.7 (17)	15.7 (16)			
IA	18.0 (27)	10.4 (13)				13.7 (14)	10.8 (11)			
Other benign nodules^b^	18.0 (27)	19.2 (24)				19.6 (20)	20.6 (21)			
Surgical duration (min)^a^	85.00 (64.25–131.25)	65.00 (50.00–81.00)	0.596	−6.173	0.000	80 (60.00–100.00)	70.00 (50.00–91.00)	0.200	−1.669	0.095
Intraoperative bleeding volume (ml)^a^	10.0 (10.0–30.0)	10.00 (5.00–10.00)	0.344	−6.530	0.000	10.00 (10.00–20.00)	10.00 (5.00–20.00)	0.067	−0.667	0.505

Normally distributed data were presented as means (standard deviation), non-normally distributed data were represented as medians (interquartile ranges: first quartile–third quartile) and categorical data were presented as % (*n*).

a Maximum lesion diameter, surgical duration, intraoperative bleeding volume: non-normally distributed data.

b Other benign nodules: inflammatory lesions, fibrous hyperplasia and other non-tumour lesions.

AAH: atypical adenomatous hyperplasia; AIS: adenocarcinoma *in situ*; FEV1: forced expiratory volume in 1 s; IA: invasive adenocarcinoma; MIA: minimally invasive adenocarcinoma; MVV: maximal voluntary ventilation; SMDs: standardized mean difference.

### Post-PSM inter-group comparison of postoperative observation indicators

In the DT group, the postoperative mean total drainage was 178.19 ml (SD: 413.02), and the mean drainage duration was 1.37 days (SD: 1.60). In this group, chest tube removal was performed on postoperative day 1 in 89.2% (91/102) of the patients and on postoperative day 2∼3 in 8.8% (9/102) of the patients. Only 2.0% (2/102) of the patients required postoperative CTD for >3 days due to air leaks, and were resolved by CTD.

Postoperative intrathoracic bleeding occurred in 2.0% (2/102) of the patients in the DT group, and the symptoms disappeared following proper treatment with medications (Snake venom thrombin, 1 unit, administered intravenously once). In the NT group, persistent air leak were observed in 2.0% (2/102) of the patients and were effectively treated by chest tube insertion through the second intercostal space in the midclavicular line. Despite the presence of postoperative complications, none of the patients in either group required a second surgery. The postoperative complication rates were not significantly different between the 2 groups (*P *>* *0.05).

The highest POPS ranged between 1 and 3 in both groups. But it was significantly worse in the DT group than in the NT group [2.31 (SD: 0.76) vs 2.02 (SD: 0.81), respectively; *P* = 0.008]. The postoperative LOS was significantly shorter in the NT group than in the DT group [3.00 (IQRs: 2.00–4.00) days vs 2.00 (IQRs: 100–3.00) days, *P* < 0.001]. The total in‐hospital expense was also significantly lower in the NT group than in the DT group [33283.74 (IQRs: 27098.61–46718.56) yuan vs 26598.67 (IQRs: 22965.14–29933.67) yuan; *P* < 0.001].

The chest DR performed 1 week after discharge revealed that 1.0% (1/102) of the patients in the DT and NT groups developed pleural effusion, respectively; the symptoms improved after puncture and aspiration in both groups. In the NT group, 1.0% (1/102) of the patients experienced pulmonary infection, which improved following the oral administration of moxifloxacin hydrochloride (0.4 g, once a day). The 2 groups did not exhibit a significant difference in the rate of complications at 1 week after discharge (*P *>* *0.05).

The follow‐up chest CT performed 1 month after discharge to assess the recovery status of the thoracic cavity revealed that neither group developed complications. See Table 2.

**Table 2:** Postoperative and follow-up characteristics of the DT and NT groups

Characteristics	DT group (*n* = 102)	NT group (*n* = 102)	*t*/Z/χ^2^	*P*-value
Total drainage volume (ml)^a^	65.00 (28.75–132.50)	N/A	N/A	N/A
Chest tube duration (days)^a^	1.00 (1.00–1.00)	N/A	N/A	N/A
Highest postoperative pain score	2.31 (0.76)	2.02 (0.81)	−2.682	0.008
Postoperative complication, % (*n*)	5.0 (5)	3.0 (3)	2.020	0.568
Air leaks	2.0 (2)	2.0 (2)		
Incision fat liquefaction	1.0 (1)	1 (1)		
Intrathoracic bleeding	2.0 (2)	0		
Postoperative length of stay (days)^a^	3.00 (2.00–4.00)	2.00 (1.00–3.00)	−4.547	0.000
In‐hospital expense (RMB)^a^	33283.74 (27098.61–46718.56)	26598.67 (22965.14–29933.67)	−5.810	0.000
Follow‐up findings at 1 week after surgery, % (*n*)			1.391	0.499
No complications	99.0 (101)	98.0 (100)		
Pleural effusion	1.0 (1)	1.0 (1)		
Pulmonary infection	0	1.0 (1)		
Follow‐up findings at 1 month after surgery	No complications	No complications	N/A	N/A

Normally distributed data were presented as means (standard deviation), non-normally distributed data were represented as medians (interquartile ranges: first quartile–third quartile) and categorical data were presented as % (*n*).

a Total drainage volume, Chest tube duration, postoperative length of stay, in‐hospital expense: non-normally distributed data.

## DISCUSSION

A fair compromise between postoperative safety and rapid postoperative recovery is always a consideration in patients undergoing thoracoscopic pulmonary wedge resection with CTD. Faster recovery should never come at the expense of postoperative safety, which is the highest priority, and implementing postoperative CTD is a consequential consideration, given that patient safety is at stake. To date, studies have evaluated alternative postoperative drainage approaches such as single chest tube instead of dual CTD, the use of a smaller chest tube (≤12 Fr) instead of a larger one (≥20 Fr) and the omission of postoperative CTD [7–18]. Despite the small number of reported cases, current literature provides preliminary evidence supporting the safety of omitting postoperative CTD. However, the outcomes of omitting CTD following thoracic surgery remain unclear. Intraoperative insertion of a small‐bore chest tube that is removed on postoperative LOS 1, i.e. 1-day postoperative CTD, not only allows for the postoperative drainage of fluid and air from the thoracic cavity and the close observation of air leaks and intrathoracic bleeding but also reduces the impact of postoperative CTD on postoperative recovery, therefore creating a desirable compromise between postoperative safety and fast recovery. From 2018 through 2022, surgeons in our centre have performed thoracoscopic pulmonary wedge resection by routinely inserting a 12-Fr chest tube or by not leaving a chest tube for postoperative drainage. While postoperative CTD was preferred in 2018 and 2020, thoracic surgery without postoperative CTD has become the choice of treatment since 2021 in a setting where the chest tube was removed on postoperative LOS 1 in patients requiring postoperative drainage. In the present retrospective study, we compared U‐VATS wedge with and without postoperative CTD and analysed the differences between these approaches in terms of patient safety and postoperative recovery.

In the present study, we utilized PSM to eliminate the significant differences in baseline characteristics between the 2 groups, including sex, age, lung function (FEV1, MVV), heart function (EF), pulmonary nodule size, number of simultaneously resected pulmonary nodules and pulmonary nodule location. Additionally, we utilized PSM to reduce the statistical bias introduced by CTD due to prolonged surgical duration and excessive intraoperative bleeding. The 2 groups exhibited no significant differences in these aspects after the screening of patients with criteria such as prolonged surgical duration and excessive intraoperative bleeding.

Post‐PSM analysis of the postoperative observation indicators suggested that U‐VATS wedge without postoperative CTD did not increase the postoperative complication rate. In the NT group, only 2 of the 102 (2.0%) patients required bedside closed drainage because of postoperative air leaks. In the DT group, 2 (2.0%), 2 (2.0%) and 1 (1.0%) patients experienced postoperative air leaks, intrathoracic bleeding and poor wound healing, respectively. It is unclear whether postoperative CTD interferes with tissue healing and increases the risk of postoperative air leaks and the risk of intrathoracic bleeding or bleeding from the wound in the chest wall. The rate of complications 1 week after discharge did not differ significantly between the 2 groups, implying that thoracoscopic pulmonary wedge resection without postoperative CTD had at least an acceptable safety profile. Nonetheless, closer observation and surveillance are essential in postoperative care. For instance, bedside chest X-ray examination should be performed within 6 h after surgery for the early detection and treatment of pleural effusion and pneumothorax.

Our analyses also revealed more mild postoperative pain, shorter postoperative LOS and lower total in‐hospital expenses in the NT group compared with the DT group, indicating the physical and financial benefits of the no-postoperative-drainage policy. In fact, without CDT patients can be discharged on the day of the surgery, but the vast majority of patients choose to stay in the hospital for observation one night to ensure that they are comfortable before being discharged. Therefore, we usually discharge patients who without CDT on the day after the surgery. Otherwise, the postoperative LOS and hospitalization costs would less in the NT group.

Simply put, as an uncomplicated procedure, thoracoscopic pulmonary wedge resection only inflicts 2 wounds, one on the chest wall and the other at the resection margin. The thoracoscopic cutting stapler is safe and reliable for the resection and suture of the pulmonary tissue; this approach avoids the improper placement of staples at the resection margin and entails a low risk of postoperative air leaks and bleeding from the resection margin. With a surgical wound of 2–3 cm in size, massive postoperative haemorrhage in the thoracic cavity is unlikely as long as proper haemostasis is achieved. In fact, chest drainage at the end of surgery serves as a test for air leakage as it indicates the air leak based on the formation of bubbles in the drainage system and allows for the further reduction in the risk of postoperative pneumothorax by sealing the air leak at the time of detection. In such cases, postoperative CTD is unnecessary.

### Limitations

We acknowledge the limitations of the present study. First, this is a single-centre retrospective study, and the thoracoscopic pulmonary wedge resection procedure is likely to differ among surgeons and hospitals. Therefore, multicentre prospective studies are warranted to investigate the potential differences in postoperative complication rates in relation to the omission of postoperative CDT among different hospitals. Second, during the period of change in the concept of placing a chest tube or not, the doctor's decision to place a chest tube has a certain subjectivity, which will affect the conclusion of the study. However, since this is a retrospective study, it is not possible to completely rule out this effect. Third, the presence of postoperative air leaks observed in both groups, despite the low incidence, requires further investigation to determine whether these leaks are associated with marginal leakage or an unnoticed postoperative rupture of pulmonary bullae. Lastly, even after PSM, the standardized mean differences for gender, actual MVV/pre-MVV, and surgical duration were still >0.1, implying a suboptimal balance in PSM.

## CONCLUSIONS

The current study findings provide preliminary evidence that U-VATS wedge without postoperative CTD was safe and feasible. The no-postoperative-drainage policy did not increase the risk of complications but significantly reduced the postoperative pain, the length of hospital stay and the in‐hospital expenses. Multicentre studies, including larger cohorts, are warranted for stronger evidence.

## ETHICAL STATEMENT

The authors are accountable for all aspects of the work in ensuring that questions related to the accuracy or integrity of any part of the work are appropriately investigated and resolved. The study was conducted in accordance with the Declaration of Helsinki (as revised in 2013). The study was approved by the Ethics Committee of the Xiamen Humanity Hospital of Fujian Medical University (No. HAXM-EMC-20221017-001-01) and individual consent for this retrospective analysis was waived.

**Conflict of interest:** none declared.

## Data Availability

The data underlying this article will be shared on reasonable request to the corresponding author.

## ABBREVIATIONS

CTD: Chest tube drainage
DR: Digital radiograph
EF: Ejection fraction
FEV1: Forced expiratory volume in 1 s
IQRs: Interquartile ranges
LOS: Length of stay
MVV: Maximal voluntary ventilation
POPS: Postoperative pain score
PSM: Propensity score matching
SD: Standard deviation
U-VATS: Uniportal video‐assisted thoracoscopic pulmonary wedge resection
